# SAPHO syndrome: a review

**DOI:** 10.1007/s11832-014-0627-7

**Published:** 2015-01-14

**Authors:** Iva Rukavina

**Affiliations:** Department of Paediatrics, University Hospital Centre Zagreb, Kišpatićeva 12, 10000 Zagreb, Croatia

**Keywords:** SAPHO, CRMO, Hyperostosis, Osteitis, Arthritis

## Abstract

**Introduction:**

Synovitis–acne–pustulosis–hyperostosis–osteitis (SAPHO) is an acronym for various osteoarticular and dermatological manifestations that can appear in the same patient. It is a rare syndrome, but since its awareness has increased, there have been more and more such reports in the literature.

**Aims:**

The objectives of this review are to summarize the current state of knowledge on pediatric and adult-onset SAPHO syndrome, and to discuss treatment strategies that should be considered.

**Results:**

The SAPHO syndrome can affect patients of any age, and its etiology is still not known. The syndrome has its cognizable radiological characteristics that are most important in making the diagnosis. There are several diagnostic criteria as well, but they need further validation. No standard treatment protocols are available and current treatment options are not evidenced-based due to the rarity of the syndrome. Therapy is empirical and aimed at easing pain and modifying the inflammatory process. It includes nonsteroidal anti-inflammatory drugs (NSAIDs) as the first-line agents. Antibiotics, corticosteroids, disease-modifying anti-rheumatic drugs, biologicals targeting tumor necrosis factor alpha or interleukin-1, and bisphosphonates have all been used with variable success. Surgery is reserved to treat complications. Even though it is a disease with good long-term prognosis, its treatment remains a challenge and the results are known to be disappointing, especially with the skin component of the disease.

**Conclusion:**

It is expected that these patients present at the time of diagnosis and the treatment should be as early, effective, and safe as possible in order to prevent osteoarticular progression and to limit the adverse events associated with pharmacological drugs.

## Introduction

The SAPHO (synovitis–acne–pustulosis–hyperostosis–osteitis) syndrome was first introduced by the rheumatologist Chamot in 1987, and it is characterized by a combination of skin and osteoarticular manifestations. The term attempts to comprise numerous names that have been used in the literature for the last 50 years, describing the above-mentioned characteristics. Some of those names are bilateral clavicular osteomyelitis with palmar and plantar pustulosis, inter-sterno-costo-clavicular ossification, subacute and chronic symmetric osteomyelitis, arthro-osteitis associated with a follicular occlusive triad, sternoclavicular hyperostosis, nonbacterial osteitis, pustulotic arthro-osteitis, chronic recurrent multifocal osteomyelitis (CRMO), Koehler’s disease, pyogenic sterile arthritis, acquired hyperostosis syndrome, or spondyloarthritis hyperostotica pustulo-psoriatica [1–4]. CRMO will be discussed more in detail in this review as well, due to the great confusion that these two terms generate in the literature. Numerous authors have suggested that CRMO and SAPHO lie along the same clinical spectrum. Some believe that CRMO is the pediatric presentation of SAPHO, even though there are some rare descriptions of SAPHO seen in children and seldom in adolescents, as well as descriptions of CRMO in adults. It seems that the differentiating clinical feature is mainly in the localization of inflammation: in pediatric CRMO, the extremities are more often affected, whereas in SAPHO, the axial skeleton with costosternoclavicular region is the focus [5–9].

SAPHO is considered a rare disease and sufficient data on its prevalence are unavailable. It is predominantly found in patients with average ages of 30 and 50 years [10]. Hayem et al. [11] reviewed 120 cases of SAPHO and revealed that there is a female predominance among patients younger than 30 years old at the beginning of the disease. Despite all of this, there is a considerable number of reports on children who suffer from SAPHO, and, today, it is considered that it can evolve at any age [1]. The youngest described patient was only 15 months old [12]. According to some authors, the annual prevalence is estimated at 1/10,000 in Caucasians [6, 13] or 0.00144/100,000 in Japanese [14–16].

The clinical presentation is heterogeneous and patients may, therefore, present to different specialists. SAPHO is well known to dermatologists and rheumatologists, but there are only a few reports in the orthopedic literature. Since the disease can evolve at any age, it is important to present such literature to clinicians dealing with children, especially since various manifestations (pustulosis and osteitis) do not necessarily coincide. Recognizing the disease in time will prevent osteoarticular progression. Otherwise, patients can suffer deformity, loss of function, and increasing pain, which might require wide resections.

A MEDLINE search using SAPHO, SAPHO syndrome, and chronic recurrent multifocal osteomyelitis as keywords was performed and, further, relevant articles from retrieved references were extracted.

The objectives of this review article are to review the etiology, presentation, diagnosis, treatment, pathogenesis, and genetics of the syndrome and to raise awareness of this entity.

## Etiology

Whether the SAPHO syndrome represents a clinical entity by itself, should be considered a subset within the family of spondyloarthropathies (due to the frequent affliction of the axial skeleton, enthesitis, and inflammatory bowel diseases), or be considered a variant of another rheumatic disease (i.e., psoriatic arthritis) is still unknown [2].

The pathogenesis of SAPHO is probably multifactorial and it involves a combination of genetic, infectious, and immunological components.

The published data show that HLA-B27 is more frequent in SAPHO, but spondyloarthropathies overlap with SAPHO, and statistical analyses performed on those cohorts resulted in a higher proportion of HLA-B27 SAPHO patients. Therefore, it is no wonder that other studies refute these data and showed that there are no relations between SAPHO and HLA-B27. According to some authors, there is a positive connection with HLA 39 and HLA 61 [4, 17, 18]. Due to some similarities between SAPHO and other autoinflammatory syndromes with a genetic basis and due to familial clustering, several other genes are being studied. Researchers have discovered that genes which seem to play a role in the SAPHO syndrome are located in the chromosome 18: LPIN2 and NOD2. LPIN2 encodes lipin 2, which is involved in modulating apoptosis of polymorphonuclear cells, and mutations of the NOD2 gene may lead to an abnormal immune response to bacterial peptidoglycans via activation of the proinflammatory transcription factor nuclear factor kappa B [19].

There are also hypotheses of infectious disease, suggesting that bone lesions are caused by a low-virulence pathogen [2, 13]. Different types of pathogens were isolated from different bone sites and pustules in the skin, including *Staphylococcus aureus* [20], *Haemophilus parainfluenzae*, and *Actinomyces*, as well as *Treponema pallidum*, *Veillonella*, and *Eikenella* [21]. The most important is *Propionibacterium acnes*, which is identified more often, but positive cultures can only be seen in a small number of total bone biopsy specimens. The largest number of *P. acnes*-positive biopsy specimens was proved by Assmann and Simon [2] in their study of 21 SAPHO patients, where 67 % of them were positive. This infectious hypothesis is supported by increased levels of circulating IgA in these patients and there is also evidence that intra-articular injection of inactivated *P. acnes* in rats can cause erosive joint lesions. On the other hand, according to some of the latest considerations, since *P. acnes* is found in only two-thirds of biopsies at most and the treatment with antibiotics is effective only for as long as it is taken, it is considered that SAPHO cannot be classified among infections, even due to latent organisms [22–24].

There are various reports on immune system dysfunction in SAPHO [25–29]. According to some of them, humoral immune response is hyperactive and in others, it is hypoactive. This is similar to the cell-mediated immune response that has been reported as normal or hyperactive; total immune system impairment has been reported as well [28]. Hurtado-Nedelec et al. showed that SAPHO is characterized by elevated IL-8 and IL-18 levels. They had not detect any autoantibodies among their SAPHO patients, including rheumatoid factor, anti-CCP2, or antinuclear antibodies. IL-8 and TNFα production by purified polymorphonuclear leukocytes (PMN) were elevated in these patients compared to the controls, but the oxidative burst and IL-18 production were normal. They also showed that, after 28 days of etanercept therapy, PMN, IL-8, and TNFα production was downregulated and TNFα plasma levels were increased [30]. Assman and Simon [2] have shown that the proinflammatory response observed in SAPHO is mediated by the ability of *P. acnes* to trigger interleukin IL-1, IL-8, and IL-18 and TNFα release by monocytes, keratinocytes, sebocytes, and dendritic cells.

After all, the most probable hypothesis about the etiology of SAPHO is that it is caused by autoimmune reactions in genetically predisposed organisms, triggered by some infectious agent [2].

Regarding the pathogenesis of CRMO, infectious and autoimmune theories have been suggested, but none of them have been proven. There is a significant genetic contribution to pathogenesis and besides LPIN2, several other genes have to be mentioned. A susceptibility locus on chromosome 18q21.3–18q22 affecting the proline-serine-threonine phosphatase interacting protein 2 (PSTPIP2) has been reported [31] in a small German CRMO cohort. Furthermore, the disorder is also associated with polymorphisms of the IL-10 promoter and mutation of IL1RN causing deficiency of the interleukin-1 receptor antagonist (DIRA), an autosomal recessive disorder that presents with CRMO during the neonatal period [32].

## Clinical features

SAPHO syndrome should be suspected in patients who present with osteoarticular and/or certain dermatological clinical manifestations.

**Fig. 1:**
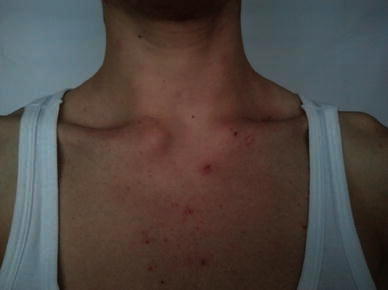
Bilateral sternoclavicular joint edema in the SAPHO patient

Osteoarticular manifestations involve osteitis, hyperostosis, synovitis, arthropathy, and enthesopathy that present with pain, tenderness, and sometimes swelling over the affected areas and fever. Osteitis is the inflammation of bone, which may involve the cortex and the medullary cavity. Hyperostosis reflects excessive bone growth and may result in enthesopathic new bone formation and joint fusion (Fig. 1). Synovitis mostly manifests as nonerosive oligoarthritis of larger joints. Joint involvement can be primary arthritis or an extension of the osteitis adjacent to the articular structures. Arthritis has been reported in up to 92.5 % of SAPHO cases. The axial skeleton is involved in 91 % and the peripheral joints in 36 % of cases. Besides sternocostal and sternoclavicular joints, which are the most commonly affected, it mainly affects the sacroiliac or hip joints, knees, and ankles. For anterior chest wall disease, three stages have been described (Table 1). The costoclavicular ligament is involved in 48 % of cases, and it is considered a decisive early finding in SAPHO [7, 32, 33].

**Table 1:** SAPHO anterior chest wall staging [26]

I. Costoclavicular ligament, may be a primary enthesopathy
II. Extension to sternoclavicular joint with sclerosis of the medial clavicle, first rib, and adjacent sternum, and sclerotic hypertrophy of costal cartilage
III. Osteosclerosis, hyperostosis, and bony hypertrophy of the medial ends of the clavicles, sternum, and upper ribs, with arthritis in the adjacent joints

**Fig. 2:**
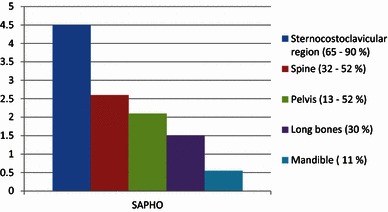
Percentage distribution of arthritis in the body (SAPHO/CRMO)

The smallest number of cases in the literature are based on temporomandibular joint involvement [11, 13, 34, 35]. The percentage distribution of arthritis in various parts of the body is demonstrated schematically in Fig. 2.

Soft tissue surrounding joints and bones can be affected as well. It may be misinterpreted as a neoplastic or lymphatic mass [7, 36], and, although rare, the soft tissue swelling can lead to serious complications, such as thoracic outlet syndrome [11, 36–38].

Enthesopathy can lead to ligament ossification, which can result in the development of bony bridging across joints.

CRMO is an aseptic inflammatory disorder clinically characterized with insidious onset of bone lesions with pain and swelling that is often worse at night, with or without fever. Swelling and warmth can occur over the affected areas. It is most commonly found in the metaphyseal regions of long bones of the lower extremities. Some other sites, such as the clavicules, vertebral bodies, mandible, pelvis, and small bones of the hands and feet, have been shown to be affected as well. Involvement is multifocal, usually unilateral, and it can be accompanied by skin lesions (most often, palmoplantar pustulosis and psoriasis have been described) [32, 39]. As stated earlier, some investigators believe that CRMO is the pediatric presentation of SAPHO, but it seems that the differentiating clinical feature is mainly in the localization of inflammation: in pediatric CRMO patients, the extremities are more often affected and in SAPHO patients, the axial skeleton with costosternoclavicular region is the focus [5].

Typical skin lesions seen in SAPHO patients include palmoplantar pustulosis (PPP) and severe acne [40]. Acne can manifest as acne conglobata, acne fulminans, or hidradenitis suppurativa. Women more often develop PPP and men show severe forms of acne. Pyoderma gangrenosum is the other less frequent manifestation and different forms of psoriasis have also been described [41], as well as Sweet’s syndrome and Sneddon–Wilkinson disease [42]. Skin lesions may vary in severity and may precede (in 50 % of the cases), follow, or occur simultaneously with the onset of arthritis [11]. Usually, the time interval between the onset of skin and osteoarticular manifestations is <2 years [43], but an interval of 38 years has been recorded in the literature [11, 44]. Sonozaki et al. [45] showed that skin lesions precede or follow the onset of osteoarticular lesions within 2 years in about 70 % of patients, while Hayem et al. [11] showed that the skin manifestations anteceded or presented at the same time as the skeletal manifestations in 68 % of their cohort. Dermatological manifestations are known to be resistant to therapy and quite often have a chronic, protracted course.

## Radiological features

**Fig. 3:**
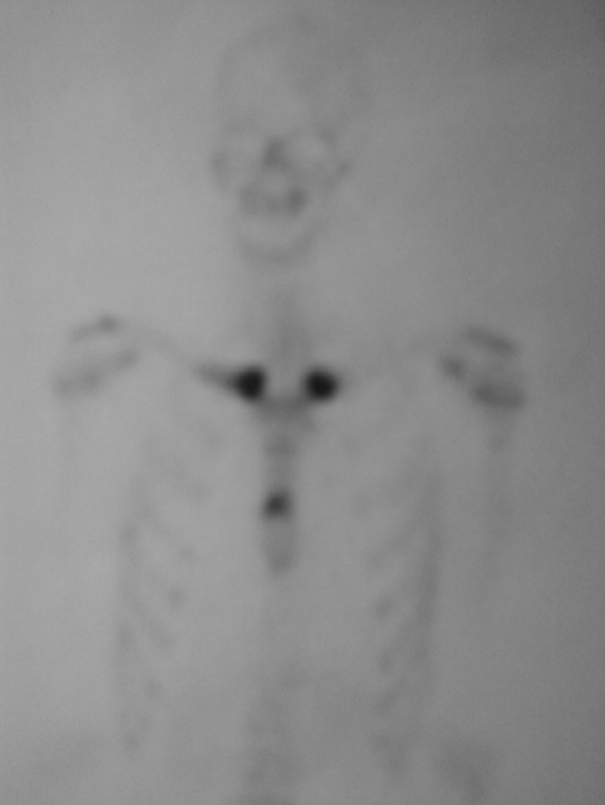
Scintigraphy findings show intensive uptake of the radiopharmaceutical technetium-99m at the sternoclavicular joints and sternum, which represent a “bull’s head“ sign

Radiographs may show expanded bone, sclerosis and osteolysis, periosteal reaction, or enthesopathic new bone formation. Bone scintigraphy delineates increased uptake in affected bone and may reveal asymptomatic disease or abnormalities not apparent on radiographs. The advantage of scintigraphy is the demonstration of multiple sites of involvement, so it is helpful for the elimination of malignancy or infection. Symmetric uptake in the sternoclavicular region with a typical “bull‘s head” appearance shown in bone scintigraphy is characteristic of the SAPHO syndrome (Fig. 3) [46]. It was first described by Freyschmidt and Sternberg [47] but, even though it is considered to be pathognomonic, it is not a very sensitive indicator of SAPHO.

Magnetic resonance imaging (MRI) will also detect occult lesions, may show findings not seen on plain radiographs, and provide information about soft tissues.

Characteristic radiographic findings are hyperostosis and osteitis. Hyperostosis is radiographically seen as diffuse thickening of the periosteum, cortex, and endosteum, with narrowing of the medullary canal [47]. Both are characterized by increased bone sclerosis [35, 39].

In the early stages, the disease usually manifests as an osteolytic process. As healing progresses, the lytic/sclerotic picture is produced. Characteristic features of osteitis and hyperostosis become more apparent with time [35].

Joint involvement is characterized by arthritis, with joint space narrowing and, sometimes, erosions. There might be periarticular osteopenia. Ligamentous ossifications can be observed as well [32, 37].

Several spine lesions have been described regarding this syndrome, and they include vertebral body corner lesions, nonspecific spondylodiscitis and osteodestructive lesions seen in adults and children, and osteosclerotic vertebral lesions, paravertebral ossification, and sacroiliitis seen in adults.

The term “corner lesion” describes focal cortical erosion at one of the vertebral body corners, which is usually seen in adults. Nonspecific spondylodiscitis is seen as focal erosive changes with sclerosing remodeling of the vertebral end plates, usually anteriorly located at the discovertebral junction. This can be seen in up to 32 % of cases, and single and multiple levels may be found [35]. Takigawa et al. [14] observed nonconsecutive and consecutive multilevel lesions, both at a proportion of 38 %. It may be painful for many weeks but, usually, with time, it becomes asymptomatic. Rarely it is a cause of neurological complications or deformity [35].

Osteodestructive lesions include osteolytic vertebral lesions, usually limited to one vertebrae, with a variable degree of collapse. Collapse may induce kyphosis, spinal canal stenosis, and spinal cord injury. If it is quite marked, it can present as a vertebra plana in children, which is not characteristic of an adult population [14]. Sacroiliitis can be seen and it is usually unilateral. Ankylosis may be present as well, and it is usually connected with the relief of pain [7, 38, 48].

Affection of the long bones is commonly seen among children. Predominantly, the metadiaphyses are affected, especially the distal femur, and proximal and distal tibia. Radiographically, it may manifest as lytic lesions, sclerotic or mixed lesions, and periosteal reaction may eventually develop. MRI is the technique of choice in young patients suspected of SAPHO/CRMO, particularly due to the lack of radiation requirements and its sensitivity in detecting early subclinical lesions. It is seen as bone marrow edema, which shows up as hypointense on T1 and hyperintense on T2 signals in the affected metaphysis. As the disease progresses, hypointense T1 and T2 signals in the medullary space and cortex represent medullary sclerosis and cortical thickening [17]. Lesions are usually multiple and often symmetrical. Involvement of the adjacent epiphysis and altered bone growth are rare [17, 35].

Many of the radiological manifestations of the disease can be seen on plain radiographs. It is important to emphasize that radiographs made during the first 3 months of the disease course are normal in 80 % of cases and all patients had abnormal radiographs at the end of follow-up [38]. Similar findings were shown by Fritz et al. [49]. They found that the sensitivity of conventional radiography in the early stages of the disease is 13 % and, compared to MRI, it shows only 16 % of the lesions seen on MRI. For identifying subclinical foci, whole-body scintigraphy or whole-body MRI is very useful. Actually, if initial radiographs are negative and disease is suspected, bone scintigraphy is used as the next step to detect occult inflammatory lesions and clinically suspected localizations. Because of increased cost, the use of whole-body MRI is recommended for indeterminate cases, monitoring of disease activity, and for better delineation of soft tissue changes. Intravenous contrast will highlight abscesses and other soft tissue changes that may be associated with more aggressive conditions [17]. It should be kept in mind that imaging procedures cannot accurately distinguish among SAPHO/CRMO, malignancy, and osteomyelitis, and such findings should always be interpreted within other clinical and laboratory parameters.

## Laboratory tests

There are no laboratory tests that are diagnostic of SAPHO. They can be normal or may show elevated inflammatory markers, such as erythrocyte sedimentation rate (ESR), C-reactive protein (CRP), and elevated levels of components of complements C3 and C4. Mild leukocytosis and mild anemia were observed as well. Compared to healthy controls, these patients have elevated levels of immunoglobulin A [2, 50]. A study searching for some specific antibody profiles for those patients has been conducted recently, but, unfortunately, without any success. Hurtado-Nedelec et al. [30] showed significantly increased levels of IgA in their cohort of 29 SAPHO patients, while the levels of IgM and IgG were normal. This information can possibly be used as an additional tool in making the diagnosis, but further investigations need to be done. Also, some studies exhibit correlation with B39 and B61 [18].

## Histopathological findings

Osteitis refers to bone inflammation and appears histopathologically as sterile inflammatory infiltrate [3]. Early during the disease course, the predominant finding is PMN infiltrate. In the intermediate stage, the infiltrate is composed primarily of mononuclear cells and in the late stage, bone trabeculae are enlarged and sclerotic, with an increased number of osteocytes and marrow fibrosis. Skin biopsy of the affected skin shows neutrophilic pseudo-abscesses [30]. SAPHO and CRMO lesions are histologically identical [40].

## Diagnostic criteria

There are several published diagnostic criteria for SAPHO and the presence of only one of the inclusion criteria is sufficient for making the diagnosis. The criteria suggested by Kahn and the other by Benhamou are the most frequently mentioned. All of them are preliminary and need further validation (please see Tables 2, 3, and 4). With regard to all of them, it can be said that the criteria made by Kahn and modified in 2003 seems to be the most precise. Even though the existence of such criteria is very helpful in making the diagnosis, it is very doubtful as to whether bone and joint involvement associated with chronic bowel diseases (which is one of the inclusion criteria) can be classified as SAPHO syndrome, since arthropathies associated with inflammatory bowel disease are included in the EULAR/ILAR criteria for juvenile idiopathic arthritis (JIA) [51, 52].

**Table 2:** Diagnostic criteria proposed by Kahn for SAPHO syndrome diagnosis, 1994 [41]

1. Chronic recurrent multifocal sterile and axial osteomyelitis, with or without dermatosis
2. Acute, subacute, or chronic arthritis associated with palmoplantar pustulosis, pustulous psoriasis, or severe acne
3. Any sterile osteitis associated with palmoplantar pustulosis, pustulous psoriasis, or severe acne

**Table 3:** Diagnostic criteria proposed by Kahn for SAPHO syndrome diagnosis, modified in 2003 (from Kahn; American College of Rheumatology 67th Annual Scientific Meeting, October 2003) [6]

*Inclusion*
Bone–joint involvement associated with PPP and psoriasis vulgaris
Bone–joint involvement associated with severe acne
Isolated sterile^a^ hyperostosis/osteitis (adults)
Chronic recurrent multifocal osteomyelitis (children)
Bone–joint involvement associated with chronic bowel diseases
*Exclusion*
Infectious osteitis
Tumoral conditions of the bone
Noninflammatory condensing lesions of the bone

^a^Exception: growth of *Propionibacterium acnes*

**Table 4:** Diagnostic criteria proposed by Benhamou for SAPHO syndrome diagnosis [29]

1. Osteoarticular manifestations in severe acne
2. Osteoarticular manifestations in palmoplantar pustulosis
3. Hyperostosis with or without dermatosis and
4. Recurrent multifocal chronic osteomyelitis involving the axial or peripheral skeleton, with or without dermatosis

Furthermore, it should be discussed whether CRMO is the pediatric presentation of SAPHO or an entity by itself. For those reasons, these criteria need further modifications.

## Diagnosis and differential diagnosis

The diagnosis is usually made by a rheumatologist, who will consult with a dermatologist to treat the skin component of the disease. Making a diagnosis is challenging because not all symptoms are always apparent or present at the same time, or some may be subtle. In making the diagnosis, the above-mentioned clinical, radiological, and laboratory characteristics, as well as diagnostic criteria, are used. Regarding differential diagnosis, early during the disease course, infectious (osteomyelitis) and neoplastic etiology must be excluded. Tumors with local extension should be considered—thyroid cancer, lymphoma or osteosarcoma, metastatic breast, prostate cancer, and neuroblastoma [17]. Psoriatic arthritis with axial skeleton manifestation and pustular psoriasis, a special subgroup of psoriatic disease, can be the cause of diagnostic dilemma. Radiographic signs of osteitis with hyperostosis are not often seen in psoriatic arthritis [53]. Furthermore, differential diagnosis includes Paget’s disease (genetic disease with increased bone turnover, repeated fractures and deformities, markedly elevated level of alkaline phosphatase, and radiographs revealing characteristic mosaic pattern, both osteolysis and osteosclerosis) and Sweet’s syndrome (neutrophilic dermatosis with elevated inflammatory markers that can be accompanied with aching joints). When the clavicle is affected, Tietze’s syndrome (swelling of the costal cartilages, mostly in adults, rare in children) and avascular necrosis of the clavicular epiphysis are considered as well. Regarding differential diagnosis in the pediatric age group, it also includes Ewing’s sarcoma, histiocytosis, Majeed syndrome, or DIRA. Majeed syndrome is an autosomal recessive (AR) disorder that presents with early-onset CRMO and dyserythropoietic anemia. DIRA is an AR disorder that manifests with CRMO in the neonatal period with generalized pustulosis, osteitis, periostitis, and systemic inflammation [17, 32, 54]. Differential diagnosis of SAPHO and CRMO is shown in Table 5.

**Table 5:** Differential diagnosis of SAPHO/CRMO [17, 32, 54]

Osteomyelitis
Lymphoma
Osteosarcoma
Metastatic cancer
Psoriatic arthritis
Paget’s disease
Tietze’s syndrome
Sweet’s syndrome
*Only in children*
DIRA
Majeed syndrome
Ewing’s sarcoma
Histiocytosis

Finally, it should always be kept in mind that SAPHO and CRMO are diagnosed by exclusion. When only one site is involved in the absence of skin lesions, making the diagnosis can be difficult and biopsy may be needed. Sterile osteitis (little or no medullary change) is one of the major characteristics of this syndrome, but the diagnosis can never be done by histological results alone, and the advantage of biopsy is just to exclude other diagnoses [24, 46].

## Treatment

Because to the variety of clinical presentations, the treatment of SAPHO syndrome remains a challenge and outcomes are known to be disappointing, especially with the skin component of the disease. There have been no randomized controlled trials on the effectiveness of various therapies, but nonsteroidal anti-inflammatory drugs (NSAIDs) are generally considered as the first-line treatment option [4]. Antimicrobial therapy is useful in patients with positive biopsy cultures, but it has little or no effect in others. Successful treatment has been reported for doxycycline, azithromycin, sulfamethoxazole/trimethoprim, and clindamycin [20, 55]. Azithromycin acts not only as an antimicrobial, but also as an anti-inflammatory and immunomodulatory drug, and Schilling and Wagner suggest the simultaneous usage of azithromycin together with calcitonin (osteotropic drug) [56]. Other treatment options include colchicine, corticosteroids, bisphosphonates, and disease-modifying agents, such as methotrexate, sulfasalazine, and anti-TNFα therapy. Bisphosphonates act by inhibiting bone resorption and turnover, and by possible anti-inflammatory activity that suppresses the production of IL-1, IL-6, and TNFα [57]. They have no effect on skin lesions. Local corticosteroid injections have also been tried, but this treatment modality has a significant effect only on osteitis lesions [53]. Some authors used corticosteroids orally and, in that case, they will act on both skeletal and skin manifestations. Dermatologists use topical corticosteroids, psoralen plus ultraviolet A (PUVA) photochemotherapy, and retinoids [58]. Disease-modifying agents are only indicated when symptoms persist for at least 4 weeks, despite adequate NSAID therapy. There is increasing evidence of anti-TNFα usage in the treatment of such patients. Case reports and case series on TNFα blockade often demonstrate a marked improvement in the clinical picture, regardless of whether or not this treatment is permanently effective. The most often published cases in the literature are about the use of infliximab in these patients. Usually, 5 mg/kg at weeks 0, 2, and 6 followed by a 6–8-week interval has been used, just like that used in spondyloarthropathies. Lower doses of infliximab and reduction in the duration of intervals have been tested, but it has been noted that decreased infusion intervals like in spondyloarthropathies and lower dosages cannot maintain the remission of disease [58]. Both skeletal and cutaneous lesions responded well in most of the described cases, with exception of PPP, which sometimes failed to respond. In some cases, infliximab induced exacerbation of skin manifestation. Arias-Santiago et al. [59] suggested adalimumab as a possible alternative therapy in such cases, and there are also reports on the successful treatment of SAPHO with etanercept and the IL-1 receptor antagonist anakinra. Anakinra appeared to be helpful in five out of six SAPHO patients, two of which previously failed to respond to TNF blockers [60]. Autologous bone transplantation using microvascular flaps is applied as an experimental treatment procedure [15].

Physiotherapy can always be used as an additional treatment for osteoarticular manifestations. Surgery is considered for patients whose condition has failed to respond to all other therapeutic interventions [61]. Wide resections are reserved to treat complications when patients develop deformity or loss of function with pain [15]. There are several reports in the literature about the surgical treatment of such patients; for example, resection of the medial clavicle or the sternoclavicular joint, which seemed to provide variable improvement in pain, although some authors report no improvement with this intervention [54]. Furthermore, mandibular involvement has been treated with minor surgical procedures, such as decortications and curettage, but extensive extirpation of the cortical jaw was done as well [62].

## Clinical course and conclusion

Except for a minority of patients who have a self-limited course, most of them have either relapsing–remitting course or chronic indolent pattern. Over the long term, rheumatic manifestations in most patients show little progression [11]. Maugars et al. [38] revealed that, after an average follow-up of around 12 years, 53 % of patients develop disease at new sites.

Colina et al. [48] identified that female sex, anterior chest wall involvement, peripheral arthritis, skin lesions, and high inflammatory parameters at first presentation are related to the chronic course of the disease.

SAPHO is rare, but as awareness increases, it is being reported more often. It should be suspected when evaluating patients with lytic, sclerotic, or hyperostotic bone lesions and pain.

This paper is an attempt to increase awareness about SAPHO syndrome among orthopedic pediatric surgeons and prompt recognition will help avoid unnecessary examinations, biopsies, surgical treatments, antibiotic therapy, or possible physical and psychological impairments associated with the disease, especially among children. It is important to remember that the skin manifestations and bony involvement may not be present at the same time, and it would be best to refer suspected SAPHO patients to the rheumatologist/dermatologist. Further examination and randomized controlled trials need to be done in order to better understand the disease, as well as to aid the development and establishment of adequate therapies.
